# Aerosolized miR-138-5p and miR-200c targets PD-L1 for lung cancer prevention

**DOI:** 10.3389/fimmu.2023.1166951

**Published:** 2023-07-13

**Authors:** Qi Zhang, Jing Pan, Donghai Xiong, Junjun Zheng, Kristi N. McPherson, Sangbeom Lee, Mofei Huang, Yitian Xu, Shu-hsia Chen, Yian Wang, Lea Hildebrandt Ruiz, Ming You

**Affiliations:** ^1^ Center for Cancer Prevention, Houston Methodist Cancer Center, Houston Methodist Research Institute, Houston, TX, United States; ^2^ Center for Immunotherapy Research, Houston Methodist Cancer Center, Houston Methodist Research Institute, Houston, TX, United States; ^3^ Department of Chemical Engineering, The University of Texas at Austin, Austin, TX, United States

**Keywords:** MicroRNAs, miR-138-5p, MiR-200c, aerosol delivery, lung carcinogenesis, tumor immune microenvironment

## Abstract

The development of chemopreventive strategies with the ability to prevent the progression of lung lesions to malignant cancers would reduce the mortality and morbidity resulting from this deadly disease. Delivery of microRNA (miRNA) by inhalation is a novel method for lung cancer prevention. In this study, we investigated the combined efficacy of aerosolized miR-138-5p and miR-200c miRNA mimics in lung cancer prevention. Combination of the two miRNAs inhibited Benzo(a)pyrene (B((a))P)-induced lung adenomas and N-nitroso-tris-chloroethylurea (NTCU)-induced lung squamous cell carcinomas with no detectable side effects. Using single-cell RNA sequencing (scRNA-seq) and imaging mass cytometry (IMC), we found that both miRNAs inhibited programmed cell death ligand 1 (PD-L1) expression. Our flow cytometry results showed that aerosolized delivery of combined miRNAs increased CD4+ and CD8+ T cells and reduced the expression of programmed cell death protein 1 (PD-1) and T-regulatory cells. Our results demonstrated that the delivery of aerosolized microRNAs targeting PD-L1 can be highly effective in preventing lung cancer development and progression in mice.

## Introduction

The two major histopathological types of lung cancer are non-small cell lung cancer (NSCLC) and small cell lung cancer (SCLC). About 85% of lung cancer is histologically classified as NSCLC including adenocarcinoma and squamous cell carcinoma (SCC) (1). Chemoprevention is an important approach to reduce this deadly disease.

One of the major advances in translational studies of lung cancer chemoprevention is the development of mouse NSCLC models of the major subtypes of lung cancer. These models enable identification of novel chemopreventive agents with strong efficacy specifically against lung cancer subtypes. Multiple carcinogens have been identified to establish lung adenocarcinoma (2). B(a)P is one of the well-studied tobacco-specific carcinogen, it induces mouse lung tumors develop key KRAS mutations [G12C (56%), G12V (25%) and G12D (19%)] similar to those observed in mutant KRAS human lung cancer (3, 4). In comparison, a few mouse SCC models were validated for use in preclinical cancer chemoprevention studies. We have developed a mouse SCC model induced by carcinogen N-nitroso-trischloroethylurea (NTCU) that has similar histopathological features and keratin staining to human lung SCC (5), and been widely used worldwide in chemoprevention, carcinogenesis, and imaging studies (6, 7). In the current study, we evaluated the chemopreventive efficacy in these two carcinogen-induced primary lung tumor models that represent the major subtypes of lung cancer.

microRNAs (miRNAs) are small non-coding RNA molecules that regulate the expression of human genes. MiRNAs can inhibit gene expression by binding to complementary regions of mRNA and either blocking translation or degrading mRNA. MiRNAs target lots of mRNAs and influence the expression of multiple genes. Underexpressed miRNAs in cancer can be functionally classified as tumor suppressors, while the overexpressed miRNAs act as oncogenes. Tumor-suppressive miRNAs could be useful in treating tumors (8, 9). However, there are challenges to the use of miRNAs including poor cell penetration, inefficient delivery to the desired target tissue, and undesired toxicities (10). Recently, MRX34 was evaluated for its efficacy against melanoma; however, the drug was withdrawn due to serious adverse effects in a Phase 1 clinical trial (https://clinicaltrials.gov/ct2/show/NCT02862145) (11).

Inhaled medications have been available for many years for treating lung diseases (12). As compared to systemic administration, drugs delivered directly to the lungs *via* inhalation can result in better efficacy at lower doses with decreased toxicity (9, 13–15). Aerosol delivery of therapeutic drugs for lung cancer in humans has been reported to be efficacious with minimal systemic distribution of the therapeutic agents (16). One novel approach for lung cancer chemoprevention is the use of aerosolized tumor suppressor miRNAs to decrease side effects and improve efficacy. In our previous studies, we successfully used a synthetic miRNA along with neutral lipid emulsion without any side effects (17).

miR-138 is consistently among the most decreased miRNA in human cancers including lung, colorectal, ovarian, nasopharyngeal, pancreatic, head/neck squamous cell carcinoma, and many others (18). The well-characterized miRNA-200 (miR-200) family functions as tumor suppressors that have been found to be involved in cancer development and metastasis (19). The expression of miRNA was decreased in lung cancer, pancreatic cancer, and bladder cancer (20–22). Both miR-200 and miR-138 were shown to target the expression of PD-L1 (23, 24). We found the expression of these two miRNAs is significantly lower in lung cancer tissue than in adjacent normal tissues after comparing lung adenocarcinoma and SCC tumor samples with normal lung tissues from the TCGA project (**Supplementary Figure S1**). Therefore, we hypothesize that the combination of miR-138 and miR-200 miRNAs *via* aerosolized delivery may present as highly effective agents for chemoprevention of lung adenocarcinoma and squamous cell carcinoma in mouse models without systemic toxicity.

In this study, the efficacy of aerosolized miR-200c, miR-138-5p, and their combination in lung cancer prevention was investigated. We found that the combination treatment exhibited striking tumor inhibition in both the B(a)P-induced lung adenoma and NTCU-induced lung squamous cell carcinoma mouse models without side effects. Using single-cell RNA sequencing (scRNA-seq), we found that aerosolized miRNA treatment increased the proportion of cytotoxic CD8+ T cells mediating antitumor function. Using imaging mass cytometry (IMC) in a syngraft mouse model, we found that tumor inhibitory effects of the miRNAs likely involve decreased PD-L1 expression. Together, our results indicate that miRNAs promote antitumor immunity *in vivo* and that treatment with aerosolized miRNA mimics is a promising approach for lung cancer prevention.

## Materials and methods

### Reagents and animals

B(a)P, tricaprylin, acetone, miR-200c, and miR-138-5p mimic were purchased from Sigma Chemical Co. (St. Louis, MO). Neutral Lipid Emulsion (NLE) was purchased from BIOO Scientific (Austin, TX). NTCU was purchased from Toronto Research Chemicals, Inc. B(a)P dissolved in tricaprylin was prepared fresh before administration to animals. NTCU dissolved in acetone was prepared fresh before use. A/J mice and SV129 mice were purchased from Jackson Laboratory. Swiss mice were purchased from Charles River Laboratories.

### Aerosol procedure

The miRNA mimic (16 nmol/ml in NLE) was atomized into droplets using our custom-made collision-type atomizer. The total exposure time of mice to miRNA was 10 min per treatment, twice per week. These inhalation exposures were given using a custom-built nose-only exposure chamber. The effluent aerosol was discharged from an opening at the bottom of the chamber. Mice were exposed one at a time to the aerosol by placing their noses into the cone of the apparatus (17). The diameter of particles was determined to be in the nano–micrometer range (**Supplementary Figure S2**), which is favorable for mouse inhalation. The size distribution of drug aerosol produced by the atomizer was determined by a Scanning Electrical Mobility Spectrometer (SEMS, Brechtel model 2002) consisting of a differential mobility analyzer (DMA) and a condensation particle counter (CPC). To calculate the geometric mean diameter (GMD) and geometric standard deviation (GSD), a lognormal distribution was fit to the particle number size distribution data (25).

Mass Mean Diameter (MMD) was calculated from:


$$D_{V}^{3}=\frac{V_{t}}{N_{t}}\left(\frac{6}{\pi }\right)$$

where D_V_ is the volume mean diameter, which is the same as the MMD assuming a constant density, N_t_ is the total number concentration, and V_t_ is the total volume concentration.

### Expression of miR-138-5p and miR-200c in human lung cancer tissues

Data files containing the scRNA-seq raw count data of the two TCGA human lung cancer patient cohorts (lung adenocarcinomas and lung squamous cell carcinomas) were downloaded using the software pipeline- TCGA-assembler 2 (26). Batch effects were adjusted using the R package RUVSeq. Data normalization and differential expression analysis were performed using the statistical algorithms in the statistical R package edgeR. FDR-corrected *P* values of less than 0.05 were considered as significantly regulated miRNAs.

### Efficacy of miRNA mimics in the B(a)P-induced lung cancer model

To characterize the efficacy of miRNA on suppressing lung carcinogenesis, the B(a)P-induced lung tumor model in A/J mice was used. Six-week-old female A/J mice were injected with the chemical inducer B((a)P (single i.p. dose, 100 mg/kg in 0.2 ml tricaprylin). One week after the B(a)P injection, mice were randomized into four groups: 1) scrambled miRNA control group; 2) miR-200c miRNA group; 3) miR-138-5P miRNA group; 4) Combination group. Mice were treated with aerosol twice per week. All mice in each test group were exposed at the same time to the agent aerosol by placing their noses into the cone of the mouse channel. The body weights of the mice were measured every week for the duration of treatments. After 22 weeks of the miRNA treatment, mice were euthanized, serum was collected, and analyzed by Marshfield Labs for glucose, and liver function enzymes alanine transaminase (ALT), aspartate aminotransferase (AST). Lungs were fixed and evaluated under a dissecting microscope to obtain surface tumor count and individual tumor diameter. Tumor volume was calculated based on the following formula: V = 4πr^3^/3.

### scRNA-seq analysis of mouse lung tumors

For scRNA-seq, B((a)P-induced primary lung tumors were harvested and pooled from each mouse at the end of the study, then minced into 1-2 mm^3^ pieces and digested at 37°C for 20 min with mouse tumor dissociation buffer (Miltenyi Biotec, CA) to generate single-cell suspensions. Red blood cells were lysed with Ammonium-Chloride-Potassium (ACK) buffer, single-cell suspensions were stained with 7-AAD and CD45 on ice for 30 min, and CD45- and CD45+ populations were sorted by flow cytometry. For single-cell library preparation, flow-sorted CD45- or CD45+ cells were pelleted by centrifugation at 300 g for 5 min and counted manually using a Neubauer Chamber. Approximately 1.6 × 10^4^ cells were loaded onto the 10x Chromium controller per the manufacturer’s instructions. The scRNA-seq libraries were generated by Chromium single cell 3’ v3 reagent Kits (10x Genomics) and sequenced using NextSeq 500/550 high output kits v2 (150 cycles) (Illumina) according to the manufacturer’s protocols.

### Binding site analysis

Binding sites were predicted using miRWalk software (27, 28), as previously described (17). To filter the candidate binding sites, the stringent criteria of 1) binding probability >0.9 and 2) free energy<−15 (kJ mol−1) were used.

### scRNA-seq data analysis

Raw sequencing data were de-multiplexed and converted to gene-barcode matrices using the Cell Ranger (version 2.2.0) mkfastq and count functions, respectively (10x Genomics). The mouse reference genome mm10 was used for alignment. Data were further analyzed in R (version 3.4.0) using Seurat (version 3). The number of genes detected per cell, the number of unique molecular identifiers (UMIs), and the percent of mitochondrial genes were plotted, and outliers were removed (cells that expressed less than 200 and more than 2,500 genes) to filter out doublets (two single cells) and dead cells. Differences in the number of UMIs and percent of mitochondrial reads were regressed out. Raw UMI counts were normalized and log-transformed. To analyze the sequenced CD45-negative cells from mouse lung tumors, we utilized the Seurat R package3 to perform fine clustering of the single cells (29, 30). The gene expression data from all single cells were aligned and projected in a 2-dimensional space through uniform manifold approximation and projection (UMAP) to allow identification of the cell populations among the CD45- or CD45+ cells.

Differential gene expression analysis of scRNA-seq data was performed as follows: before differential expression analysis, the computational imputation of zero values was performed to correct for the influence of dropout events (i.e. failure in detecting expressed genes due to low sequencing depth of single cells). We utilized computational methods described previously (31) to perform imputation and other data processing procedures. Specifically, gene expression levels were quantified using metric log2 (TPM+1). Transcripts per million (TPM) is a normalization method for RNA-seq and should be read as “for every 1,000,000 RNA molecules in the RNA-seq sample, x came from this gene/transcript”. Missing gene expression values were imputed using the scImpute algorithm with default parameters and TPM values and gene lengths (for a gene associated with multiple transcripts, the length of the longest transcript was used) as the input. Imputation was only applied to genes with dropout rates (i.e. the fraction of cells in which the corresponding gene has zero expression value) larger than 50% to avoid over-imputation. The imputated scRNA-seq data were then subjected to differential expression analysis using the DEsingle program to assess differences between the treatment and scrambled control groups. A list of the overall differential expression results was used as input into the GSEAPreranked tool implemented in the GSEA (Gene Set Enrichment Analysis) program. Hallmark gene sets listed in the MSigDB (molecular signatures database: https://www.gsea-msigdb.org/gsea/msigdb/index.jsp) were used to test gene expression signatures to detect the important biological processes that are affected by miRNA treatment in the mouse lung cancer model.

### Efficacy of miRNA mimics in the NTCU-induced lung cancer model

Eight-week-old Swiss mice were randomized into four groups, as described above. All animals were given NTCU through repeated skin painting (5, 32). Specifically, the dorsal skin of the mice was shaved followed by applying NTCU topically to the skin as follows: 100-microliter drops of 0.04 M for each mouse (acetone is the solvent for the NTCU), twice a week, with a 3-day interval for the duration of the study. Animals in all groups received the carcinogen, NTCU. One week after the first dose of NTCU, when mice were approximately 10 weeks old, they were treated with aerosol twice per week. After 30 weeks of miRNA treatment, mice were euthanized by CO_2_ asphyxiation. All lobes of Lungs from mice were fixed in 10% buffered formalin for histopathological analysis. Approximately 100 serial tissue sections (5-μm each) were made from the formalin-fixed lung, and 1 in every 20 sections (approximately 100 μm apart) was stained with H&E and examined histologically under a light microscope. The lesions, including invasive SCC, carcinoma in situ, and bronchial hyperplasia/metaplasia, were scored in a blinded manner from the H&E-stained sections of each lung. H&E-stained slides were then scanned with the NanoZoomer HT slide scanner (Hamamatsu Photonics).

### Imaging mass cytometry sample preparation

To characterize immune cell profile changes and validate scRNA-seq findings of PD-L1 inhibition at a post-transcriptional level within tumor regions, we used IMC to interrogate the tumor immune microenvironment (TIME). Lung tumor samples were stained with a validated mouse IMC antibody panel of 11 markers. 4-6 regions of interest (ROIs) from each sample were chosen, ablated, and scanned for further analysis. Tumor regions within each ROI were identified by referring to the neighboring H&E stained slide. All analysis was done within the tumor regions. Antibodies were labeled with metals using the MaxPar antibody conjugation kit according to the Fluidigm protocol. The metal-labeled antibodies were diluted in Candor PBS Antibody Stabilization solution (Candor Bioscience) after determining the percent yield by absorbance measurements at 280 nm for long-term storage at 4°C. This study’s antibodies and their metal conjugation information are listed in **Table S1**. Tumor sections were baked at 60°C overnight, then dewaxed in xylene and rehydrated in a graded series of alcohol (ethanol absolute, ethanol: deionized water 90:10, 80:20, 70:30, 50:50, 0:100; 10 minutes each) for IMC. Heat-induced epitope retrieval was conducted on a heat block at 95°C in sodium citrate buffer at pH 6 for 20 minutes. After immediate cooling for 20 minutes, the sections were blocked with 3% bovine serum albumin in tris-buffered saline (TBS) for 1 hour. For staining, the sections were incubated overnight at 4°C with an antibody master mix. Samples were then washed 4 times with TBS. For nuclear staining, the sections were stained with Cell-ID Intercalator (Fluidigm) for 5 minutes and washed twice with TBS. Slides were air-dried and stored at 4°C for ablation. The sections were ablated with Hyperion (Fluidigm) for data acquisition (33). IMC data were segmented by ilastik and CellProfiler. Data were processed in Histology Topography Cytometry Analysis Toolbox (HistoCAT) (34), and mean intensity values were extracted at single cell level. R scripts were used to cluster single cells by RPhenograph (35), identify cell types, and quantify cell numbers. Tumor masks for all ROIs were manually annotated in QuPath (36) by referring to the H&E staining of adjacent slides. R scripts were used to summarize the cell density of different cell phenotypes and channel average intensity only in tumors. For all samples, tumor and cellular densities were averaged across 4-6 ROIs per group.

### Testing of miRNAs in the LKR13 syngraft model

The LKR13 syngraft model was used to test the immune-related effects of miRNAs *in vivo*. The cells were trypsinized, washed with PBS, and then suspended in PBS at a concentration of 2.5 × 10^6^ cells/ml. A total of 5 × 10^5^ cells was injected into the tail vein of eight‐week‐old female SV129 mice. One week after the injection of LKR13 cells, mice were divided into 2 groups: 1) the control group; 2) the miRNA treatment group (n=5). One week after treatment, lung tumors were harvested and examined by flow cytometry analysis. To test the efficacy of aerosolized miRNA treatment in tumors with reduced PD‐L1 expression, PD‐L1 was knocked down (KD) in LKR13 cells by shRNA lentivirus (Santa Cruz Biotech). LKR13 cells or PD‐L1 KD LKR13 cells (5 × 10^5^) were injected into the tail vein of 8‐week‐old female SV129 mice. Three days after the injection of LKR13-LUC cells, mice were divided into 4 groups: 1) control group; 2) miRNA treatment group; 3) shPD‐L1 control group; 4) shPD‐L1 with let‐7b treatment group. Aerosolized miRNAs were given twice per week. Mice were imaged using the Lumina IVIS‐100 *in vivo* Imaging System (Xenogen Corporation). Regions of interest were created and measured as area flux, defined by radiance (photons per second per square centimeter per steradian).

### Flow cytometry

For immune profiling, tumors were harvested and pooled from each mouse at the end of the study, minced into 1–2 mm^3^ pieces, and digested at 37°C for 20 min with mouse tumor dissociation buffer (MiltenyiBiotec, CA) per the manufacturer’s instructions to generate single‐cell suspensions. Tumor‐infiltrating leukocytes were directly stained for flow cytometry sorting or analysis. Cells were stained with the following cell surface and viability markers: 7AAD for live/dead cells, BV786 anti‐CD45, APC eFluro780 anti‐CD3, FITC anti‐CD4, BUV396 anti‐CD8a, SB600 anti‐CD19, PE‐Cy7 anti‐CD44, APC anti‐CD62L, and PE anti‐CD25 antibodies. For intracellular cytokine staining, cells were stimulated for 4 h in RPMI medium containing Kras peptides, 10% FBS, 50 uM 2‐mercaptoethanol, 1%penicillin–streptomycin, 1× monensin, and 1× Brefeldin A (Thermofisher Sci). For Foxp3 staining, cells were washed, fixed, permeabilized, and stained with Foxp3/transcription factor eFluor450 staining buffer sets (ThermoFisher Sci) following the manufacturer’s instructions. For intracellular cytokine analysis, cells were fixed with 2% paraformaldehyde, permeabilized with 0.5% saponin, and stained with intracellular cytokine staining buffer containing Brefeldin A, APC anti‐granzyme B, PE anti‐IFN‐*γ*, and PE‐Cy7 anti‐TNF‐*α* antibody, and then analyzed by flow cytometry. T cells stained with isotype control antibodies were used as negative controls. To detect MDSCs in tumors, cells were stained with PerCP‐Cy5.5 anti‐CD45, FITC anti‐CD11b, APC anti‐CD11c, PE anti‐Ly6G, and PE‐Cy7 anti‐Ly6C Ab. Flow cytometry was conducted using an LSR Fortessa X‐20 or LSR‐II flow cytometer (Becton Dickinson). Data were analyzed using FlowJo software.

### Statistical analysis

All data are presented as mean ± standard error of the mean (SEM). Statistical analysis was performed using GraphPad Prism Software. To determine which specific groups differed from each other, we used Tukey’s “*post-hoc*” test. For comparison between 2 groups, paired Student’s *t*‐test was performed. For multiple groups (3 groups and above) comparison, one‐way ANOVA analysis was employed with Bonferroni’s post‐test. Sample sizes (n) are noted in each figure legend. *P<0.05 is considered statistically significant.

## Results

### Expression of miR-138-5p and miR-200c miRNAs in TCGA human lung adenocarcinomas and lung squamous cell carcinomas

Data files of the miRNA-seq raw count data from the two TCGA human lung cancer patient cohorts (lung adenocarcinomas and lung squamous cell carcinomas) were downloaded using the software pipeline-TCGA-assembler 2 (26). For lung adenocarcinomas, miRNA-seq data are available for 450 tumors and 45 normal lungs. For lung squamous cell carcinomas, miRNA-seq data are available for 336 tumors and 44 normal lungs. Batch effects were adjusted using the R package RUVSeq (37). Data normalization and differential expression analysis were performed using statistical algorithms in the statistical R package edgeR (38, 39). FDR-corrected *P* values of less than 0.05 were used as criteria for significantly regulated miRNAs. miR-138-5p and miR-200c expression were significantly lower in lung cancer tissue than in adjacent normal tissues with P = 0.0015 (lung adenocarcinomas) and 6.4×10^-10^ (lung squamous cell carcinoma) for miR-138-5p, and P = 5.5×10^-6^ (LUAD) and 2.6×10^-7^ (LUSC) for miR-200c (**Supplementary Figure S1**). These results indicate that miR-138-5p and miR-200c play a tumor suppressor role in both human lung adenocarcinomas and squamous cell carcinomas.

### Chemopreventive efficacy of aerosolized miRNA in B(a)P-induced lung cancer

The size distribution of aerosolized miRNA particles was measured using our custom‐built collision atomizer. The geometric median diameter (GMD) was 53.3 nm, the geometric standard deviation (GSD) was 1.6, and the mass median diameter (MMD) was 93.8 nm (**Supplementary Figure S2**). These particle sizes are suitable for mouse lung inhalation to achieve efficient deposition in mouse bronchioles, terminal bronchioles, and alveoli. The deposition dose is 18.5 µg/kg for miR-200c and 19.7 µg/kg for miR-138 µg/kg in lung. Pharmacokinetic analysis showed that the highest levels of miRNA mimic in mouse lungs were present immediately after aerosol delivery, and levels subsequently declined (**Supplementary Figure S4**).

**Figure 1:**
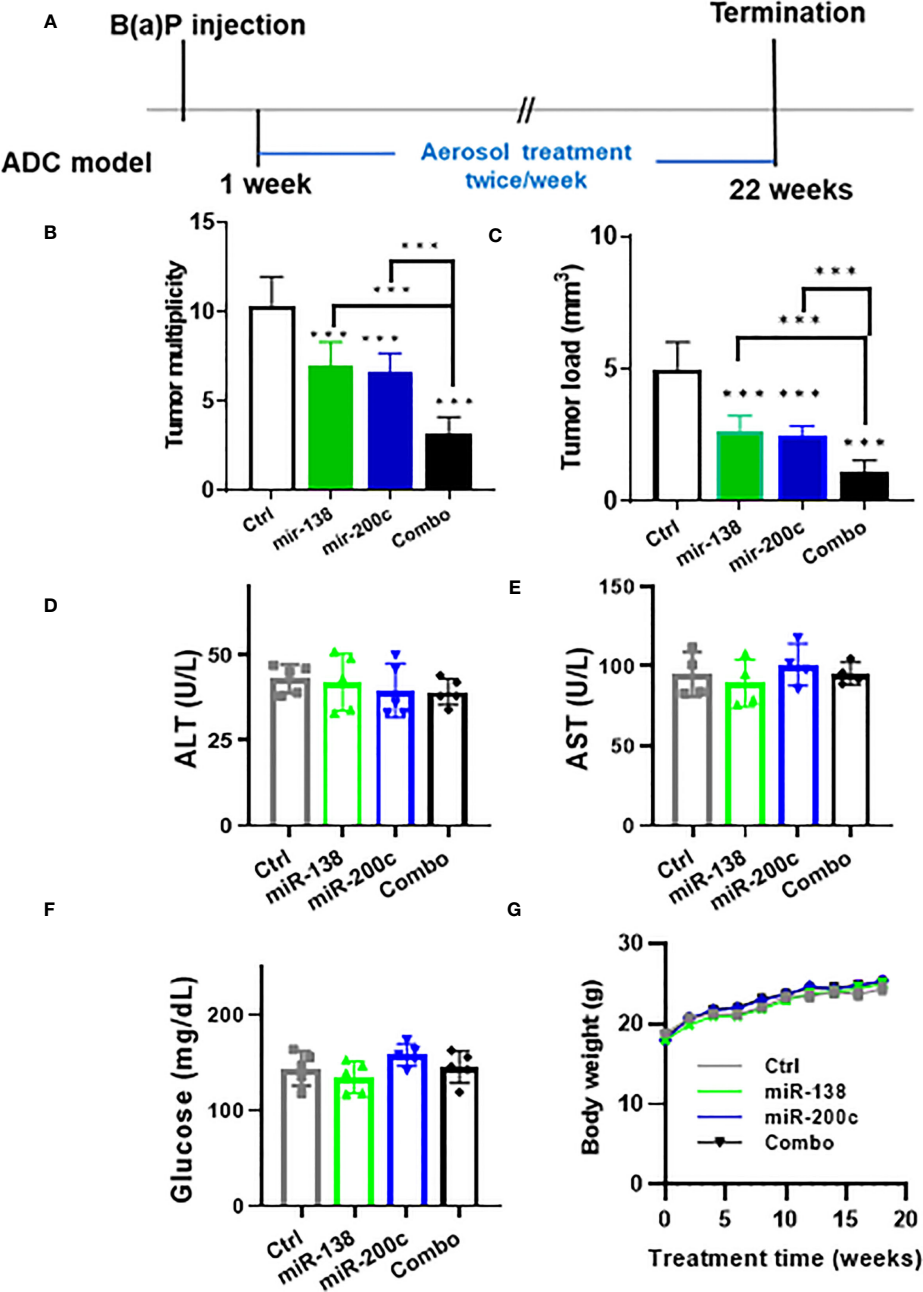
Efficacy of aerosolized miRNA in the B(*a*)P-induced lung cancer model. **(A)** Experimental the design of efficacy experiment **(B, C)** tumor multiplicity and tumor load in control and treated groups; Data presented as mean ± SEM; *n* = 8; *p< 0.001 and ***p< 0.001. *p*-values were calculated using Student’s *t*-test. **(D–F)** Potential toxicities of aerosolized miRNA were assessed by examining plasma levels of liver enzymes (*n* = 5). Plasma was collected upon termination of the experiments. **(G)** Body weights.

The efficacy of aerosolized miRNA mimics was tested in the A/J mouse B(a)P-induced lung cancer model (**Figure 1A**). In control mice, the tumor number was 10.4 ± 1.6 and the tumor load was 5.0 ± 1.1. In animals treated with mir-138-5p, the tumor number was 7.0 ± 1.3 and the tumor load was 2.7 ± 0.6 mm^3^. In animals treated with mir-200c, the tumor number was 6.6 ± 1.1 and the tumor load was 2.5 ± 0.4 mm^3^. In combination mir-138-5p/mir-200c treatment induced a better inhibitory effect on lung tumor multiplicity and tumor volume (tumor number 3.3 ± 0.9 and tumor load 1.1 ± 0.5 mm^3^, respectively). While treatments with either miR-138-5p or miR200c decreased the lung tumor load by approximately 50%, a combination of two miRNAs inhibited lung tumor load by >80% (**Figures 1B, C**), the Combo treatment showed more effective than each single miRNA. During the 22 weeks of treatment, we did not observe any changes in liver enzymes, glucose levels, or body weights (**Figures 1D-G**). Thus, aerosolized delivery of miR-138-5p and miR-200c to the lungs appears to be safe and could avoid any potential systemic side effects.

### Targeting the PD-L1/PD-1 pathway by miR-138-5p and miR-200c miRNAs

**Figure 2:**
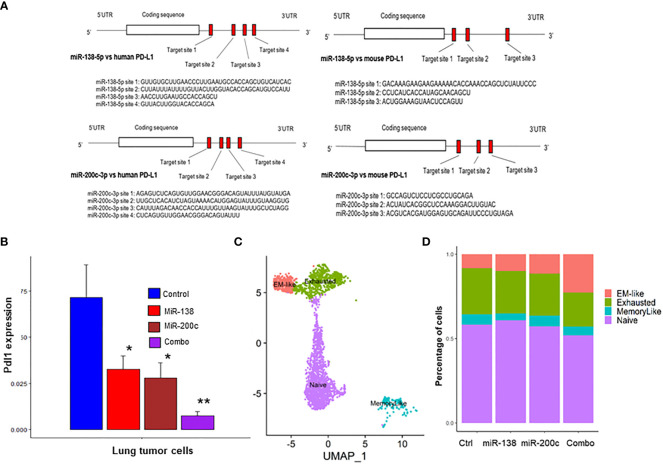
Use of scRNA-seq to identify CD8+ TIL subsets in lung tumors from A/J mice. **(A, B)** Binding assay. Suppression of PD-L1 expression in miR-200c and miR-138-treated mice. Predicted binding sites for miRNA in **(A)** human and mouse PD-L1; **(B)** Pdl1 mRNA expression levels were suppressed by miR-138, miR-200c and combo treatment in mouse lung tumor cells. **(C)** Clustering of intratumoral CD8+ T cells into four Cd8+ TIL subsets; **(D)** The combination treatment (mir-138-5p and mir-200c-3p) increased the abundance of the anti-tumor EM-like CD8+ T cells and decreased the abundance of the exhausted CD8+ T cells (n = 2; *p< 0.05 and **p< 0.01).

The binding sites for miR-138-5p and miR-200c in human and mouse PD-L1 genes were identified by miRwalk software. Four binding sites for miR-138-5p and three binding sites for miR-200c on the PD-L1 mRNA in both humans and mice were identified (**Figure 2A**). Next, we conducted scRNAseq of the lung tumors obtained from A/J mice treated with B(a)P to validate our findings. scRNA-seq was performed on lung tumors treated with miR-138-5p and miR-200c miRNAs. We found that treatments with either miR-138-5p or miR-200c decreased the expression of PD-L1 in lung tumor cells (**Figure 2B**). Interestingly, a combination of miR-138-5p and miR200c almost completely blocked the expression of PD-L1 in lung tumor cells (**Figure 2B**), presumably due to the binding of non-overlapping sites on the 3’ UTR region of the PD-L1 mRNA by miR-138-5p or miR-200c, respectively.

### Immune alterations in the microenvironment of miRNA‐treated tumors by scRNA-seq

To better understand the effects of miRNA on immune function, scRNA‐seq was performed on both tumor cells (CD45−) and immune cells (CD45+) isolated from B(a)p‐induced lung tumors in mice from the different treatment groups (**Supplementary Figure S6**). Unsupervised clustering of CD8+ tumor-infiltrating lymphocytes (TILs) by using the TILPRED program (https://github.com/carmonalab/TILPRED) identified the presence of four CD8+ TIL subsets with distinct transcriptomic profiles (**Figure 2C**). The CD8 subsets included naïve, effector‐memory (EM)‐like, memory‐like, and exhausted cells. The EM‐like (effector memory) CD8+ T cells coexpress cytotoxicity genes (Gzma, Gzmb, and Prf1) and memory genes (Lef1, Sell, and Il7r), while they lack expression of inhibitory receptors (Pdcd1, Tigit, etc.) and the exhaustion‐related transcription factor Tox. Exhausted CD8+ T cells coexpress inhibitory receptors [Pdcd1 (PD‐1), Ctla4, Entpd1 (CD39), Havcr2 (Tim3)], but lack Tcf7. Memory‐like CD8+ T cells are progenitor-exhausted cells that coexpress Pdcd1 and Tcf7 and lack Havcr2. Naive CD8+ T cells have high expression of Tcf7, Lef1, and Il7r, but no expression of cytotoxicity genes or T cell activation markers. Treatment with a single miRNA or their combination significantly increased the proportion of cytotoxic CD8+ T cells mediating antitumor function (EM-like CD8+ TILs), and conversely decreased proportions of exhausted CD8+ T cells (**Figure 2D**). These data suggest that miRNA treatment improves the overall composition of beneficial antitumor CD8+ TILs.

We then utilized the Seurat R package3 to perform fine clustering of single tumor cells (29). We tested whether miRNA treatment suppresses the cell cycle pathway in mouse lung tumors based on our scRNAseq data. Using the Seurat software and the cell cycle genes signature downloaded from the MsigDB database (https://www.gsea-msigdb.org/gsea/msigdb/), we calculated the cell cycle gene set scores of the mouse lung tumor cells across the control and miR-138-5p treated groups. The results showed that the cell cycle gene signature scores were significantly down-regulated by miR-138 treatment (**Supplementary Figure S3A**). The heatmap shows cell cycle genes that were down-regulated by the miR-138 treatment in lung tumor cells (**Supplementary Figure S3B**). We also tested whether miR-200c can suppress the EMT gene set in mouse lung tumors and found that the overall EMT signature scores (p=1.47e^-6^, **Supplementary Figure S3C**) and EMT genes (**Supplementary Figure S3D**) were down-regulated by the miR-200c treatment. These changes may be related to the antitumor efficacy of the miRNA treatment.

### Chemopreventive efficacy of aerosolized miRNA in the NTCU-induced SCC model

**Figure 3:**
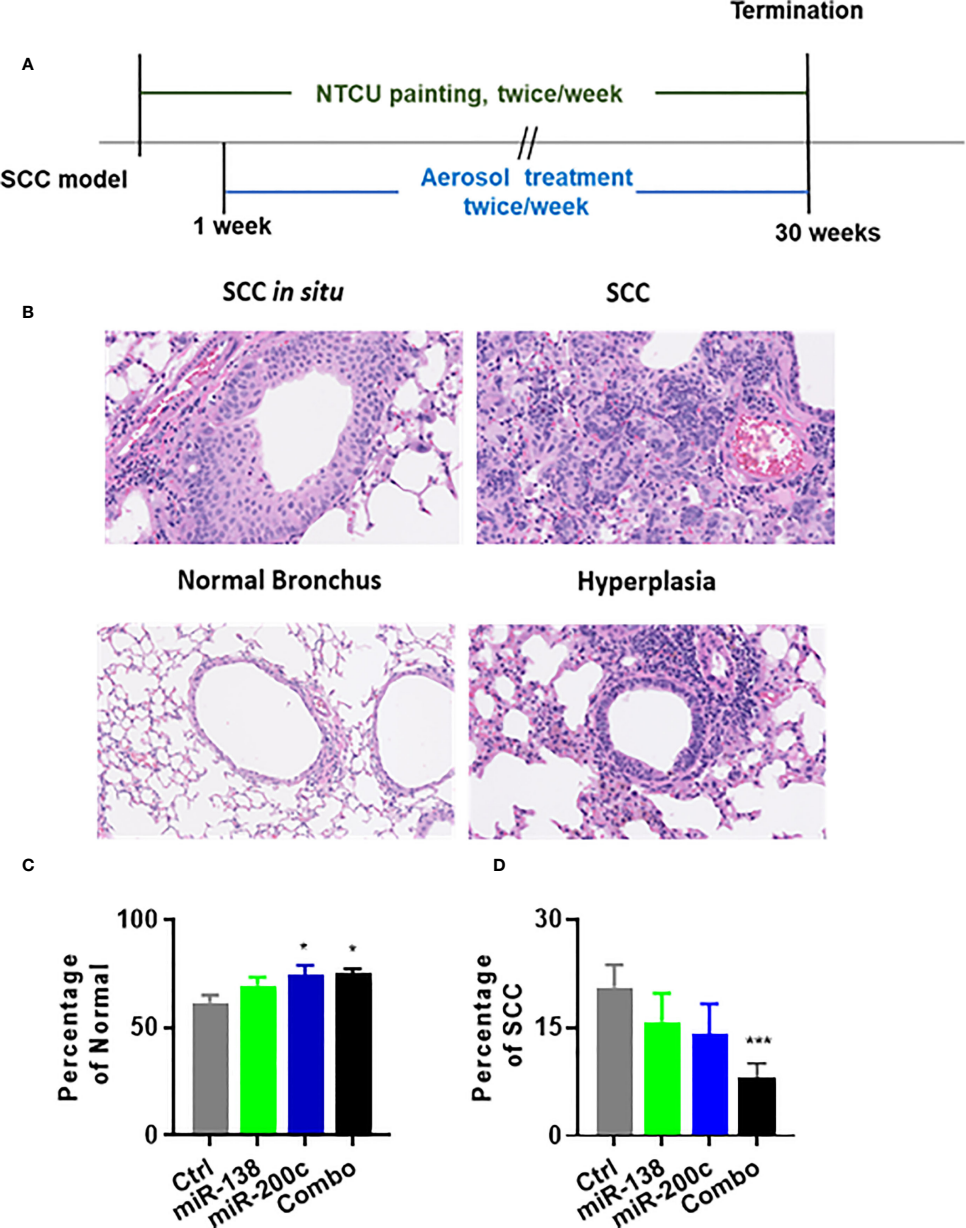
Efficacy of aerosolized miRNA on development NTCU-induced lung SCC. **(A)** Experimental design. **(B)** Representative histological images of mouse lung squamous lesions. All images are scanned by NanoZoomer system (Hamamatsu Photonics, Hamamatsu, Japan). **(C, D)** Percentage of normal bronchus and SCC in treatment groups. Statistical analysis was performed using one-way ANOVA *p<0.05. **(C)** Efficacy of miR-Let7b on the percentage of SCC development in each lung (n = 15; *p< 0.05 and ***p< 0.001).

SCC lesions induced by NTCU cannot be distinguished as clear visible nodules. Thus, serial H&E slides to analyze the number of bronchi with SCC phenotype or calculate SCC area vs. total lung area are believed to be the most suitable ways to analyze SCC lesions in this mouse model. The experimental design for assessing the efficacy of the aerosolized miRNA in the NTCU model is depicted in **Figure 3A**. Representative examples of normal bronchus and SCC are shown in **Figure 3B**. In animals treated with negative control miRNA (miR-NC), the distributions of lesions were as follows: normal bronchus was 61.7% and SCC was 20.6%. In animals treated with miR-138-5p, lesion distribution was as follows: normal bronchus was 69.4% and SCC was 11.1%. In animals treated with miR-200c, lesion distribution was as follows: normal bronchus was 74.5%, and SCC was 14.1%. Finally, in the combination group, lesion distribution was as follows: normal bronchus was 74.5%, and SCC was 8.6% (**Figures 3C, D**). Single miRNA mimic did not significantly decrease lung SCC development, while the combination did result in significantly decreased SCC. During the 30 weeks of treatment, we did not observe any changes in liver enzymes, glucose levels, or body weights (**Supplementary Figure S5**). This data indicates that aerosolized miRNAs can inhibit the progression of normal lung epithelium to lung SCC, but better effects are achieved by using a combined approach.

### Treatment with combined aerosolized miR-138-5p and miR-200c reduces PD-L1 expression and augments T cells infiltration into lung tumor tissue

**Figure 4:**
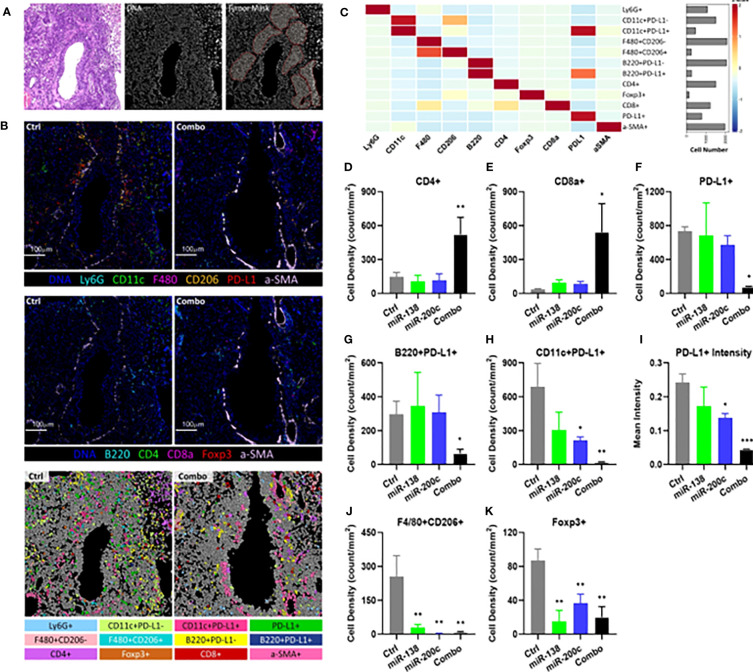
Characterization and spatial distribution of the immunological landscape in mouse SCC tumor samples using imaging mass cytometry. **(A)** Representative H&E stain (left), DNA channel (middle) and tumor mask (right) of the control sample. Scale bar=100μm. **(B)** Representative DNA, CD11c, Ly6G, F4/80, CD206, PD-L1, and αSMA (top); representative DNA, CD4, FoxP3, CD8a, B220, and αSMA images of tumors from control and combo-treated mice (middle), and cell phenotypes mapping of tumor from control and combo-treated mice (bottom). **(C)** Heatmap showing the expression of 10 lineage markers within 12 identified cell types. The heatmap was z-score transformed by column to emphasize marker expression by clusters. **(D)** Cellular densities comparisons of CD4+ T cells. **(E)** Cellular densities comparisons of CD8+ T cells. **(F)** Cellular densities comparisons of PD-L1+ cells. **(G)** B220+PD-L1+ cells (PD-L1+ B cells). **(H)** CD11c+ PD-L1+ cells (PD-L1+ DCs). **(I)** Quantification of PD-L1 intensities in TIME. **(J)** Cellular densities comparisons of F4/80+CD206+ (Type 2 macrophage). **(K)** Cellular densities comparisons of FoxP3+ cells (Tregs). For all samples, cellular densities were averaged across 4-6 images per sample from each group. The number of cells was identified per mm2 stained tumor area as the cellular densities. Significance was analyzed by unpaired two-tailed student t-test, *P< 0.05, **P< 0.01, ***P< 0.001 vs control.

Unsupervised clustering was done by Phonograph resulting in 12 cell clusters using IMC (**Figure 4A–C**; **Supplementary Figure S8**). These immune cell clusters represent dendritic cells (DCs), macrophages, T cells, and B cells, which are the major immune cell types present in TIME. Combo treatment significantly increased the CD4+ T cell (**Figure 4D**) and CD8+ T cell (**Figure 4E**) densities after treatment, whereas minimal changes were observed in single miRNA treatment groups. Besides PD-L1-expressing tumor cells, other PD-L1-expressing immune cells have also been shown to attenuate T cell function in TIME (40, 41). PD-L1 expressing DCs and B cells have also shown negative regulatory effects on T cell immunity in cancer (40). Concurrently, the densities of PD-L1 expressing cells, including PD-L1+ B cells and DCs, were significantly decreased in the lung tumor tissue from combo treatment mice (**Figures 4F–H**). Consistent with our scRNA-seq results, miR-138-5p, miR-200c and combo treatment all decreased the mean PD-L1 intensities in TIME by 28.8% (*P*=0.1062), 43.1% (*P*=0.0155) and 81.9% (*P<*0.001), respectively, as compared with tumor tissue from control mice (**Figure 4I**). Immune suppressive cells, i.e. type 2 macrophages (F4/80+CD206+) and Tregs (FoxP3+), were also significantly decreased in all treatment groups (**Figures 4J, K**).

### Combined aerosolized miR-138-5p and miR-200c treatment increased immune function and reduced Tregs in lung tumors

**Figure 5:**
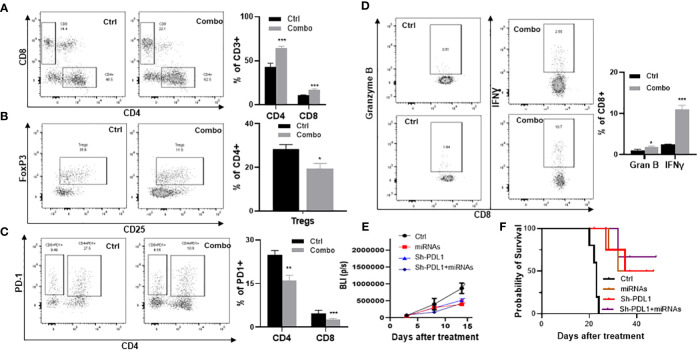
Efficacy of aerosolized miRNAs on the tumor microenvironment. **(A)** The effect of miRNAs on tumor-infiltrating CD4+ and CD8+ T cells. **(B)** The effect of miRNAs on Tregs. **(C)** The effect of miRNA on granzyme B and IFN-γ. **(D)** Percent of PD1+ cells in CD4+ and CD8+ T cell subsets. **(E)** Efficacy of miRNAs in parental LKR13 cells or LKR13 shPD-L1 KD cells. **(F)** Survival analysis of control and experimental mice. Statistical significance was calculated using log-rank test. Data presented as mean ± SEM; n=5; *p<0.05 and ***p<0.001. p-values are calculated using one-way ANOVA.

To validate our scRNA‐seq findings, mice inoculated with tumor cells from a Kras‐driven lung tumor (LKR13 cell line) were treated with the miR-138-5p/miR-200c combination, and TILs were analyzed one week later by flow cytometry for expression of specific cell surface markers. In tumor‐bearing mice one week post‐treatment, miRNA treatment led to increased accumulation of CD4+ and CD8+ T cells (p<0.001) (**Figure 5A**) and decreased intertumoral Tregs (p<0.05) (**Figure 5B**). miRNA treatment also decreased PD-1 expression on both CD4+ and CD8+ T cells (**Figure 5C**), and increased granzyme B (p<0.05) and IFN‐γ (p<0.001) producing CD8+ T cells in the lung tumors (**Figure 5D**). To further validate our observations, we used shPD-L1 lentivirus to knock down PD-L1 in LKR13 cells (PD-L1 KD LKR13 cells) (17). Aerosolized combined miRNAs were given after the mice were inoculated with tumor cells, and tumor growth was monitored by bioluminescence imaging (BLI). In LKR13 wild-type (WT)-inoculated mice, miRNA treatment inhibited tumor burden, and in PD-L1 knockdown LKR13-inoculated mice the effect of miRNA treatment was minimal (**Figure 5E**). These data suggest that the anti-tumor efficacy of combined miR-138-5p/miR-200c treatment was largely due to decreased PD-L1 expression in the tumor cells.

## Discussion

Since the identification of miR-138, several miR-138 targets have been identified, including PD-L1 and numerous cell cycle genes including cyclin D1, CDK6 & 8, and EZH2 (42–44). Increasing evidence indicates that miR-138-5p targets PD-L1 through interaction with the PD-L1 3’-untranslated region. PD-L1 and miR-138-5p levels are inversely correlated in human tumors, and miR-138-5p inhibited PD-L1 expression in mouse models (42). miR-200 miRNA has also been found to negatively regulate multiple genes involving epithelial-mesenchymal transition (45). We therefore investigated if the combination of these two miRNAs could effectively block mouse lung cancer development.

In our previous study, we demonstrated that aerosolized Let-7b significantly inhibited B(a)P‐induced lung adenoma with no detectable side effects (17). Aerosol delivery is attractive over systemic delivery since it reduces the side effects associated with systemic administration. Our data suggests that aerosolized miRNA administration is a promising approach to lung cancer prevention. In the current study, we tested the efficacy with single or combined two miRNAs by aerosol treatment. The combination significantly inhibited lung cancer in B(a)P‐induced adenoma and NTCU-induced SCC models with no apparent side effects (**Figures 1**, **3**).

Our binding sites analysis found four binding sites for miR-138-5p and three binding sites for miR-200c on the PD-L1 mRNA in both humans and mice (**Figure 2A**). Using scRNA‐seq, we conducted immune cells (CD45+) of the lung tumors obtained from A/J mice treated with B(a)P. We found that treatments with either miR-138 or miR200c decreased the expression of PD-L1 in lung tumor cells, and the combination could almost completely block the expression of PD-L1 and increase the proportion of effector memory (EM)-like CD8+ TILs (**Figure 2**). In our IMC and flow cytometry analyses. We validated that aerosolized delivery of these two miRNAs can reduce the Tregs, increase CD4+ and CD8+ T cells within tumors and induce the proliferation and effector function of tumor-specific CD8+ T cells *via* targeting PD-1/PD-L1 immune checkpoint pathway. These results suggest that these miRNAs may exert their anti-cancer effect at least partially by down-regulating PD-L1 expression (**Figures 4**, **5**). Our study also suggests that the miR-200c miRNAs negatively regulate epithelial-mesenchymal transition, and miR-138-5p negatively regulates the cell cycle pathway, which may synergistically promote antitumor efficacy in our models.

## Conclusion

In summary, our experiments demonstrate that aerosolized miRNA inhibits lung tumor growth and progression in two major lung cancer types, lung adenoma and lung SCC. miR-138 or mir-200c post-transcriptionally suppress PD-L1 and in the TIME. Our data support advancing the use of combined aerosolized miRNA for lung cancer prevention. This aerosolized miRNA could represent an exciting new approach for the prevention of lung cancer.

## Data availability statement

The data presented in the study are deposited in the NCBI BioProject database, accession number PRJNA989371.

## Ethics statement

All studies on animals were approved by the Houston Methodist Research Institute Institutional Animal Care and Use Committee (approval number: IS00006363).

## Author contributions

QZ and JP were responsible for the overall experimental design with input by JZ, KM, SL, MH, YX SC. The project was supervised by MY, JP, and SL assessed anti-cancer efficacy in animal models. JP conducted flow cytometry analysis. JZ, MH, YX and SC assessed IMC analysis. KM and LH conducted the measurements of particle size. The following were largely responsible for writing, reviewing, and editing the manuscript: QZ, JP, YW, and MY. All authors contributed to the article and approved the submitted version.
