# Supplementary material for: Aerosolized miR-138-5p and miR-200c targets PD-L1 for lung cancer prevention
Source: Front Immunol. 2023 Jul 13;14:1166951. doi: 10.3389/fimmu.2023.1166951 (PMC10372486; doi:10.3389/fimmu.2023.1166951)
Supplement: Supplementary file 9 [file Table_1.pdf]

Table s1

Table s1: The antibodies used for IMC

Marker	Metal
CD206	Nd143
Ly6G	Nd145
a-SMA	Sm147
CD11c	Sm149
Foxp3	Gd160
CD8a	Dy162
PD-L1	Dy164
B220	Er170
CD4	Yb174
F4/80	Yb176
DNA	Ir191
