# Supplementary material for: Aerosolized miR-138-5p and miR-200c targets PD-L1 for lung cancer prevention
Source: Front Immunol. 2023 Jul 13;14:1166951. doi: 10.3389/fimmu.2023.1166951 (PMC10372486; doi:10.3389/fimmu.2023.1166951)
Supplement: Supplementary file 10 [file Table_2.pdf]

Table s2: Detailed ROI analysis information for IMC results in Figure 3

ROI Name	Group	Total ROI Area (mm2)	Total Cell Number	Tumor Area (mm2)	Cell Number in Tumor Area
VC_001	Ctrl	0.490	7452	0.1262	1959
VC_002	Ctrl	0.48	7464	0.0814	1178
VC_003	Ctrl	0.49	7082	0.1444	2198
VC_004	Ctrl	0.49	7397	0.0766	1256
VC_005	Ctrl	0.50	7496	0.0685	1216
138_07_001	miR-138	0.49	5918	0.0795	881
138_07_002	miR-138	0.49	7063	0.1302	2060
138_07_003	miR-138	0.49	6321	0.1450	1879
138_07_004	miR-138	0.49	5899	0.0282	334
200_03_001	miR-200	0.49	6228	0.0872	1046
200_03_003	miR-200	0.49	6748	0.0738	1060
200_03_004	miR-200	0.49	6468	0.0683	985
200_03_005	miR-200	0.49	6616	0.0902	1168
200_03_006	miR-200	0.49	6429	0.0520	615
138_200_01_001	Combo	0.49	7341	0.1560	2544
138_200_01_002	Combo	0.49	7278	0.0924	1490
138_200_01_003	Combo	0.49	7057	0.0612	990
138_200_01_004	Combo	0.49	7589	0.1038	1726
138_200_01_005	Combo	0.49	6949	0.1093	1596
